# Influenza filaments: a graveyard of postdocs no longer?

**DOI:** 10.1038/s44318-025-00480-7

**Published:** 2025-06-09

**Authors:** Emily A Bruce

**Affiliations:** Department of Microbiology and Molecular Genetics, University of Vermont, Burlington, VT USA

**Keywords:** Microbiology, Virology & Host Pathogen Interaction

## Abstract

A recent study shows that filamentous morphology improves the ability of influenza viruses to enter host cells in the presence of neutralizing antibodies.

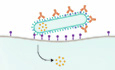

## Teaser

Influenza virions can take a variety of shapes, from the classic spherical or kidney bean morphology found in most lab-adapted strains to much longer filamentous forms that can reach over 20 nm in length. In this issue of *The EMBO Journal*, Peterl et al (2025) show that filamentous morphology improves the ability of a virion to enter host cells in the presence of neutralizing antibodies, thus shedding a new light on a role for filaments in influenza virus infection.

It has been known for decades that influenza viruses isolated from people or animals are typically filamentous, while this phenotype is lost when isolates are passed in either cells or embryonated chicken eggs, both standard ways of growing influenza virus stocks (Seladi-Schulman et al, 2013). While influenza filaments have been an object of intense interest in the field for many decades, their exact function has been curiously difficult to determine. This was not due to a lack of effort, though the topic was deemed to be “a graveyard of postdocs” by esteemed virologist Dr. Bob Lamb in the early 2000s. Work from Peterl in this issue helps to explain this finding, by showing that the filamentous shape (in viruses with identical surface glycoproteins) slows the rate of viral spread by delaying the kinetics of entry in a variety of cell types (Peterl et al, 2025). While cellular junction integrity, neuraminidase activity, and mucin did not inhibit influenza spread in a morphology dependent manner, the filamentous phenotype conferred a significant advantage in the presence of neutralizing antibodies targeting the receptor-binding hemagglutinin protein (Peterl et al, 2025, Fig. 1). This work is in agreement with other recent studies that show influenza filaments help mediate successful infection in the face of environmental pressure, including the presence of neutralizing antibodies (Li et al, 2021; Partlow et al, 2025). The use of distinct but complementary technical approaches to address this question (correlative light and electron microscopy (Peterl et al, 2025) as well as flow virometry (Partlow et al, 2025) has gone a long way to providing a robust answer to the question of what filaments are “for”.

**Figure 1:**
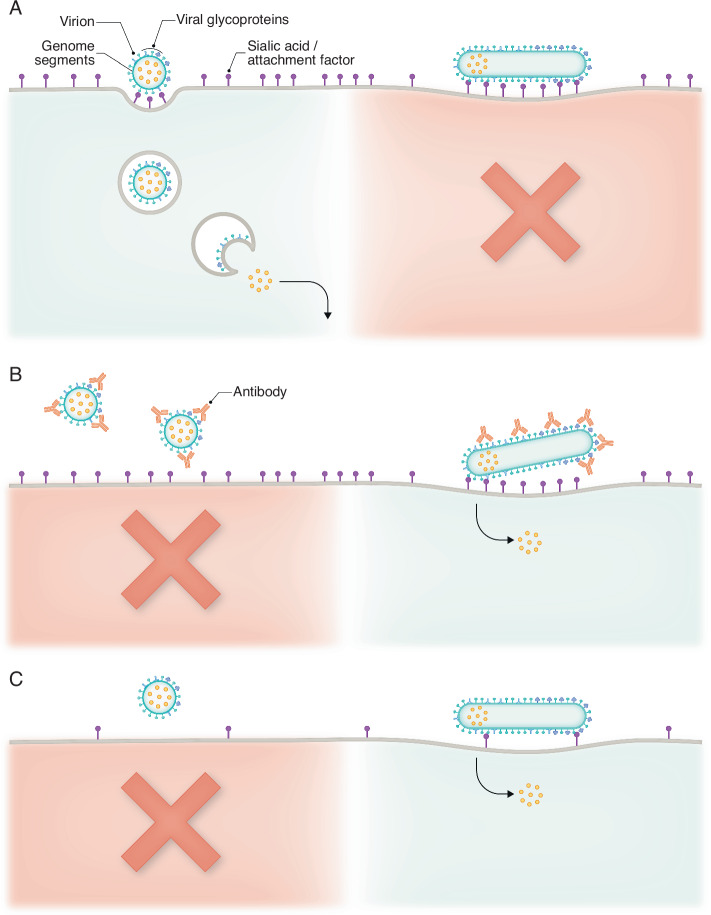
Filamentous morphology enhances influenza particle entry during adverse conditions. (**A**) Entry of spherical influenza virions is favored during situations where the molecules required for binding to the cell surface are abundant, and there are no neutralizing antibodies. This is due to the increased speed and efficiency of the pathway used by spherical virions for entry. (**B**) During conditions where neutralizing antibodies are present, the entry of filamentous virions is increased relative to spherical particles, as the increased number of HA molecules on a filament makes it more difficult to block HA/sialic acid interactions. (**C**) Filamentous virion entry is more successful in scenarios where there are few molecules on the cell surface that are compatible with binding for a given HA subtype (i.e., during the jump from one host species to another). The increased length and number of HAs give filamentous particles an advantage in binding to cells with sparse binding factors, raising the likelihood of successful fusion and entry of the genome segments.

What remains much less clear, however, is how filaments are made. The field has generated substantial data establishing the genetic determinants of filament formation, and it is clear that viral genetics contributes substantially to the phenotype. Filamentous morphology appears to be primarily determined by critical residues in both the N- and C-terminus of the Matrix 1 protein (Bourmakina and Garcia-Sastre, 2003; Elleman and Barclay, 2004; Elton et al, 2013). While M1 clearly plays a key role shaping the morphology of influenza viral particles, the complexity of the process is highlighted by the fact that motifs in the cytoplasmic tails of the glycoproteins HA and NA, as well as the ion channel M2, are known to affect particle shape in the absence of changes in M1 (Dadonaite et al, 2016).

While viral genetics explains a portion of virion morphology, several studies indicate a role for the host cell in dictating the particle shape of influenza strains that possess the ability to form filamentous particles. A functional cortical actin network is necessary for the formation of filamentous viral particles, and dysregulation of this network with jasplakinolide halts the formation of filamentous, though not spherical, particles (Roberts and Compans, 1998; Simpson-Holley et al, 2002). The Rab11 vesicular transport protein, as well as its effector protein FIP3, are also required to form filamentous viral particles (Bruce et al, 2010), likely due to a role in the trafficking of RNPs. In the absence of Rab11 (and the presumed lack of recruitment of the vRNA segments), filament extension is not seen, and virions remained stalled at the initial stages of budding (Bruce et al, 2010). Other cellular proteins, including the essential autophagy protein LC3, are also known to affect particle morphology and filament stability, though the mechanism of these interactions is less well understood (Beale et al, 2014).

While it seems clear that both viral genetics and cellular determinants contribute to influenza particle shape, it remains unclear what determines whether any given virion buds as a spherical particle or continues to extend outwards and form a filament. When examined by scanning electron microscopy, it is clear (even for a filamentous strain) that not all infected cells will produce filaments, and cells that do produce filaments generally produce spheres as well. Some prior work supports the idea that there could be a “checkpoint” required for filament extrusion, with successful delivery of the genome segments required before committing the substantial additional resources needed to produce a filament. This theory is based on the fact that the viral genome segments are reportedly seen in the distal end of budding filaments (Noda et al, 2006), and cells in which genome trafficking is disrupted display short virions with an abortive budding phenotype but fail to produce elongated particles at all (Bruce et al, 2010). Another possibility is that the difference between filaments and spheres is merely a matter of when membrane scission occurs. In this scenario, a failure to efficiently engage the budding machinery could explain the formation of filamentous virions. While influenza budding is known to be independent of the endosomal sorting complex required for transport (ESCRT) pathway (Bruce et al, 2009) used by many other enveloped viruses, the exact mechanism remains somewhat controversial.

Where current work disagrees is how fast budding virions are able to change shape in response to environmental pressures, such as passage in tissue culture or the addition of neutralizing antibodies. Using a flow virometry approach to measure subtle increases in violet light side scatter, Partlow and colleagues find that PR8 rapidly tunes its shape distribution, able to increase the proportion of filaments within hours of exposure to neutralizing antibodies (Partlow et al, 2025). In contrast, work described by Peterl and colleagues in this issue did not reveal any dramatic changes in morphology when individual WSN-M1_Udorn_ virions were observed by cryo-EM, even after five rounds of serial passage in the presence of the same neutralizing antibody used by Partlow (Peterl et al, 2025). Given the complexity of prior research, it is entirely likely that differences in strain, as well as multivalent interactions between host background and viral genetics, all combine in a multifactorial way to influence the proportion of filaments produced by each strain. Understanding how the interactions between external forces (such as antibodies) and internal forces (including specific host factors and the budding machinery) combine to determine the morphology of individual virions remains an exciting problem for the growing field of filament aficionados to address.
